# Cheoplasty and Its Results

**Published:** 1857-10

**Authors:** Alfred J. Brown

**Affiliations:** Baltimore, Md.


### ARTICLE V.

*Cheoplasty and its Results.* By Alfred J. Brown, D. D. S., Baltimore, Md.

This world has many heroes around whose brows it delights to entwine the garland of fame, and whose pathway it takes pride in strewing with flowers, but amidst them all standing pre-eminent, is he, who becomes a benefactor to his fellow man. Well and truly has it been said that "he who causes two blades of grass to grow, where but one grew before," is a benefactor of his kind, and deserves a niche in the temple of fame, around whom, all that is good and virtuous should centre, and even so is he, who in overcoming difficulties and surmounting obstacles, smooths the rugged path in the great highway of science.

To the author of Cheoplasty the highest meed of praise is due—he has nobly entered into the broad arena of science, to do battle in its holy cause, and in despite of the sneers of the unbelieving, or the malicious efforts of enemies, has in his new process won for himself imperishable honor, and brought dental mechanism to a high state of perfection. This is truly the age of progress. It is written every day and in many places. We hear it in the herculean breathings of our Atlantic steamers—in the keen whistle of our locomotives—in the hum of our telegraphs, and the advancement of science. The race of those, who hold to all the dogmas and prejudices of the past, are rapidly passing away. Old opinions as regards dental mechanism, and past prejudices against new discoveries, are swiftly taking their places upon the dusty shelf of oblivion, and new light is now shining in dark places. As one of the profession, I can heartily, and do sincerely, offer up to Dr. Blandy the incense of a grateful heart, and the time will come when the whole *profession* I trust, will "render unto Cæsar the things that are Cæsar's" and join in swelling the notes of praise that is justly due to one, who, in the onward march of science, has brought to a successful issue this invaluable invention.

Through the gloomy mist that conceals the future from the present, we can plainly discern that approaching period, when this method shall supersede all others; and for fear that some not well versed in the advantages that accrue from this system, may set me down as a visionary dreamer, I shall, in as brief a manner as possible, state a few of the reasons for my belief; a belief caused by successful results growing out of Cheoplasty in my own practice, and by my own hands. It gives us at will an accurate adaptation in all cases, of the plate to the plaster model, and when that is correct, a necessarily certain fit to the mouth. This of itself is an invaluable gift, we are no longer haunted with anxious fears as regards ultimate results, neither are we chased by the ghosts of coming accidents, for knowing the fact that this system is free in this respect from all the uncertainties which attend the old regime. We can feel that confidence which alone is necessary to confer on the practitioner the praise of skillfulness. Other important features which are attained alone by this "mode of process," is its paramount beauty—its perceptible simplicity and durability and, its high, noble and pure state of perfection, and as by degrees, the work advances in the hands of the skillful dentist, every stage of his labor secures permanently to him, the closest approach to the end, by him, so much desired. The mind of man naturally seeks to enter that path of science, where difficulties and anxieties are least to be met with, and by the members of the dental profession falling into the ranks of the supporters of Cheoplasty, they reach at once the goal of their ambition, and rest secure from all the apprehension and fears, that fall to the share of those, who still cling to old prejudices and by-gone systems; avoiding all the difficulties of the old system by an adoption of the new, with what pleasure can the operator look upon the progress of his work, and with what satisfaction can he anticipate its results. He has no uncertainty as regards its completion in a state of perfection, and thus every possibility of coming fears being avoided, he undertakes his work, with the full knowledge of the success that awaits him. And farther, we have in this new method the much to be desired facility of restoring the mouth and face to their original form, thus obtaining readily what has long been sought for, both by the patient and operator. This fact admits of no doubt, after being fully tested by myself and others, it has been found to answer every requirement, and in no instance has it failed to do all that we have ascribed to it. The ease and safety with which it can be repaired in case of accident, is also another proof of its many advantages; in fact, repairs can be made more expeditiously by Cheoplasty than that of any other process, with not the slightest risk to the piece, and when finished, the alteration is so perfect that not a trace can be seen of any such change.

Then again, possessing the same non-corrosive property as gold, and being worn with far more ease and comfort by the patient, its superiority as regards nobility is stamped at once, and exceeds in its advantages all those which have heretofore been ascribed to that metal.

In this work there are no spaces left in which food and secretions can lodge, hence for comfort, cleanliness and utility it stands unrivalled and unsurpassed; and no one on perceiving the benefits which flow from its adoption, can stand back in yielding to its author the highest meed of praise.

Lastly, but not least, with some small exceptions, we can construct it with the usual means found at the command of the profession. Requiring scarcely any additional apparatus in the laboratory of the dentist, it offers to each and every one a safe, sure and certain opportunity of testing the truths of what we have written, and he who will act and think for himself, cannot but be convinced that this process has advantages over all others, while they do not possess one single advantage over it.

In order to substantiate what we have herein advanced, we take great pleasure in laying before the reader of this communication, the following letters. It would be a useless waste of words to preface with any laudatory remarks the wisdom and energy of the author of the annexed epistle, suffice it to say, that Maryland is proud of the honor of having him among "her sons," and that all know him to have won for himself an exalted station in the temple of fame, knowing no obstacle in his onward career in the beaten pathway of science.

BALTIMORE, *July* 31, 1857.

DR. A. J. BROWN,

*Dear Sir* :—My opinion of Dr. Blandy's Process of Mounting Artificial Teeth, is very decidedly in its favor, compared with any other process. The exact adaptation of the plates to the surface and irregularities of the palatal and alveolar developments accomplished by this process, is certainly unequalled by any other process. The metal used in the process has some advantages over all others, platina, gold, silver and palladium, as I have proved by considerable experience. In my mouth all those metals have a very disagreeable black incrustation, while that used by Dr. Blandy's process remains pure and lustrous. I discover not the least taste in the metal, and on the whole, if I could have my choice of a set of teeth, mounted on either of the noble metals, or the new metal, each at the same cost, I should unhesitatingly choose the metal and the process patented by Dr. Blandy. It seems to me that this process must necessarily supersede all others. The prejudice in favor of gold, or any of what are called the "noble metals," in my opinion, based upon experience, is nothing but prejudice. For the purposes of mounting artificial teeth by the process in question, the new metal is itself the noble metal.

Respectfully,
GIDEON B. SMITH, M. D.

The next which we shall offer, is from a distinguished minister of the Protestant Episcopal Church, in this diocese, and surely with such testimony as we have here adduced, it cannot be said that we have roamed beyond the bounds of truth, in the benefits we have ascribed to it.

Princess Anne, Somerset Co., Md.
*September 8th*, 1857.

Dr. A. J. Brown,

*Dear Sir:*—I am in receipt of your favor of the 5th inst., and take pleasure in answering your inquiries respecting my experience of the advantages of Cheoplastic Dentistry.

As you well know, I have for upwards of twenty years, bestowed much attention on the various improvements, which, during that period, have been contributed by scientific men towards perfecting the Dental Art, and, as you are likewise aware, I have especially tested the qualities of the various metals and compounds which have been brought into use in the manufacture of suction plates, &c. Among these I particularly specify as articles with which I have most fully experimented, gold, silver, palladium, platina, and last of all, the valuable compound known as Cheoplasty.

Without going into any comparative estimate of the respective claims of these substances, or the advantages and disadvantages, which, upon actual trial, I found to belong to any one of the first four I have enumerated, I will briefly state the facts which have governed my mind in coming to the conclusion that Cheoplasty is justly entitled to preference over all others, and will, therefore, in all probability, supersede them all in its application to dental purposes.

1. The metal used in Dr. Blandy's process, appears from the experiment I have been able to make, to be *perfectly free from all tendency to discoloration.* The plate I have tested has been in constant use since last spring, and it does not now exhibit the slightest perceptible evidence of being in the least degree acidulated. It retains its original color as perfectly untarnished as when it first came from the crucible.

2. The perfect *tastelessness and purity* of this metal supplies another desideratum. I do not hesitate to say, after giving this article a fair and full trial, that its qualities in this respect are equal to gold, and superior to all other metals which I have tested; glass itself could not be less tasteless than I have found this new compound to be.

3. As this is an incorruptible substance, and at the same time one of great density, its *durability* and *strength* are not the least of its recommendations.

4. The last particular which I shall state, is one which is important only after the truth of the foregoing has been ascertained. I mean its *price.* The mere cheapness of an article, unless united with other claims, is of course entitled to no consideration whatever. But when an improvement is made in any department of science, having in view the comfort and well being of mankind, it receives no inconsiderable claim to public patronage in addition to that which it presents on the ground of utility, when it is found to be so cheap as to enable the poorer classes, who are by far the most numerous of our fellow creatures, to avail themselves of it. This is a recommendation which I am happy to know belongs to the Cheoplastic process, and will, unquestionably, cause it to be hailed with gladness by thousands who could not heretofore, on account of the high prices of the old metals, obtain for themselves a serviceable set of teeth. I sincerely hope and believe that many who are now suffering from the effects of imperfect mastication, will find in this valuable invention, not only an available comfort, but a real and permanent blessing.

As I have written only the result of my actual experience of the advantages of Cheoplastic Dentistry, you are at liberty to make any use of this you may deem proper.

Very truly,

Yours, &c.

JAMES MOORE,

Rector of Somerset Parish, Somerset Co., Md.

Before finally concluding our remarks, we would beg leave to mention a case, which having occurred under our own observation, and in our own family, gave to us every opportunity of testing the advantages of Dr. Blandy's mode of process. We have watched this particular case with the utmost solicitude and professional carefulness, and now that many months have elapsed since its perfection, we have not been able to discover the slightest alteration in any respect. Its silver like lustre which so adorned it when it left our hands, it still retains, in all its original brightness and purity; and for tastelessness, cleanliness, usefulness and beauty, it answers every purpose and promotes every object which we had in view at its construction.

We have thus plainly, and we hope clearly, explained the various advantages that are reaped by this process, and when we take into consideration the vast amount of time—the hours of labor, and the anxieties that are saved, we but begin to appreciate the rich blessings which follow in the path of those who become laborers in the vineyard of Cheoplasty. We can but wish, and ardently desire, that every dentist in the land, should be in the receipt of that knowledge by which his labor is shortened; his anxieties for future ills avoided, and his work standing forth a perfect masterpiece, reflecting credit alike on the operation and the operator.

And in conclusion, allow us to say, that amidst the numerous discoveries that have been brought to light by the magic wand of science, having for their aim the amelioration of the bodily comfort of mankind, Cheoplasty is entitled to the highest praise, and stands amidst them all, the noblest monument in the field of useful art.

